# Glucose-6-phosphate dehydrogenase deficiency accelerates arterial aging in diabetes

**DOI:** 10.1007/s00592-023-02118-8

**Published:** 2023-09-23

**Authors:** Angelo Scuteri, Christopher H. Morrell, Majd AlGhatrif, Marco Orru, Edoardo Fiorillo, Michele Marongiu, David Schlessinger, Francesco Cucca, Edward G. Lakatta

**Affiliations:** 1 Dipartimento Scienze Mediche e Sanita’ Pubblica, Universita’ di Cagliari, Cagliari, Italy; 2 Internal Medicine Unit, Policlinico Universitario Monserrato, AOU Cagliari, Cagliari, Italy; 3 Laboratory of Cardiovascular Sciences, National Institute on Aging Intramural Research Program, NIH, Baltimore, USA; 4 Division of Cardiology, Department of Medicine, Johns Hopkins School of Medicine, Baltimore, MD USA; 5 Istituto di Ricerca Genetica e Biomedica (IRGB), Consiglio Nazionale delle Ricerche (CNR), Lanusei, NU Italy; 6 Laboratory of Genetics, National Institute on Aging Intramural Research Program, NIH, Baltimore, USA; 7 Istituto di Ricerca Genetica e Biomedica (IRGB), Consiglio Nazionale delel Ricerche (CNR), Cagliari, Italy

**Keywords:** Aging, Arterial stiffness, Diabetes, Glucose 5 phosphate dehydrogenase, Interleukin 6, Pulse wave velocity

## Abstract

**Aims:**

High glucose levels and Glucose-6-Phosphate Dehydrogenase deficiency (G6PDd) have both tissue inflammatory effects. Here we determined whether G6PDd accelerates arterial aging (information linked stiffening) in diabetes.

**Methods:**

Plasma glucose, interleukin 6 (IL6), and arterial stiffness (indexed as carotid-femoral Pulse Wave Velocity, PWV) and red blood cell G6PD activity were assessed in a large (4448) Sardinian population.

**Results:**

Although high plasma glucose in diabetics*,* did not differ by G6DP status (178.2 ± 55.1 vs 169.0 ± 50.1 mg/dl) in G6DPd versus non-G6PDd subjects, respectively, IL6, and PWV (adjusted for age and glucose) were significantly increased in G6PDd vs non-G6PDd subjects (PWV, 8.0 ± 0.4 vs 7.2 ± 0.2 m/sec) and (IL6, 6.9 ± 5.0 vs 4.2 ± 3.0 pg/ml). In *non-diabetics*, neither fasting plasma glucose, nor IL6, nor PWV were impacted by G6PDd.

**Conclusion:**

G6PDd in diabetics is associated with increased inflammatory markers and accelerated arterial aging.

## Introduction

High glucose levels in diabetics are associated with reduced glucose-6-phosphate dehydrogenase (G6PD) activity [1–3], decreased levels of the reduced form of nicotinamide adenine dinucleotide phosphate (NADPH) cellular levels and increased pro-inflammatory markers, and accelerated arterial aging due to stiffening of large arteries [4].

Cytosolic NADP + /NADPH ratio which in fact is regulated by G6PD activity impacts on glucose metabolism, nucleotide and aromatic amino acid synthesis [5], and also regulates the level of Reactive Oxygen Species (ROS): G6PD-deficient cells are sensitive to oxidizing stimuli and more easily succumb to oxidative stress than non-G6DP deficient cells [6]. Exposure to high glucose levels decreases G6PD activity, triggering an insufficient NADPH supply and accumulation of ROS in different tissues [1–3], which activate proinflammatory pathways in G6PD deficiency [7].

Arterial aging can be evaluated as arterial stiffness (indexed as carotid-femoral Pulse Wave Velocity—PWV) [8], which captures the continuum from the early (accelerated) vascular aging to the “lower than average” arterial aging, i.e. Healthy Vascular Aging [9]; is associated with CV mortality and disability independently of conventional CV risk factors [10–12]. Additionally, we firstly reported and, then, confirmed that specific clusters of metabolic alterations are selectively associated with arterial aging [4, 13].

The island of Sardinia is characterized by the highest prevalence (~ 8–15%) in G6PD deficiency, worldwide [14]. The aim of the present study was to investigate the impact of diabetes mellitus on plasma inflammatory markers and arterial stiffness in subjects with and without G6PD deficiency in a large Sardinian population.

## Research design and methods

Red blood cell G6PD activity was determined using a quantitative assay in a 4448 (1879 men and 2569 women) participants of the SardiNIA Study aged 30 + years [15].

Blood pressure, anthropometry, metabolic risk factors, and cytokine levels were measured as previously described [4]. G6PD deficiency was defined as G6PD activity < 0.8 UI/g Hb.

Diabetes was defined according to American Diabetic Association definition [16]. Arterial Stiffness was measured as carotid-femoral Pulse Wave Velocity (PWV) [15]. Given that PWV is a highly age-associated trait, Healthy Vascular Ageing (HVA) and Early Vascular Ageing (EVA) were defined, respectively, and as a PWV value below the age-quintile specific 10th percentile, and as a PWV value above the age-quintile specific 90th percentile [9].

Using SAS University, ANCOVA analysis -including age and glucose levels as covariates- tested for interaction between diabetes mellitus and G6PD deficiency.

## Results

Diabetes was associated with greater levels of glucose and PWV (Table 1), in non-diabetics, glucose levels and PWV did not differ with those subjects with normal or deficient levels of G6PD activity. In diabetics G6PD deficient subjects had significantly greater IL6 levels, and PWV compared to diabetic subjects without G6PD deficiency. The significant (*p* < 0.05) interaction of Diabetes withG6PD deficiency for all the three variables mentioned above indicated that the impact of G6PD deficiency on glucose levels, arterial stiffness, and IL6 levels significantly differed according to the presence of diabetes. Of note, although HVA was more common in non-diabetics with G6PD deficiency, a significantly greater proportion of subjects with stiffer arteries (EVA, a PWV value above the age-quintile specific 90th percentile) was observed in diabetic subjects with G6PD deficiency.4

**Table 1:** Effect of G6PD deficiency and presence of diabetes mellitus on cardiometabolic risk profile and arterial stiffness (means ± SD)

	Controls		Diabetes		ANCOVA after adjustment for age		
	G6PD deficit NO (*n* = 3818)	G6PD deficit YES (*n* = 384)	G6PD deficit NO (*n* = 228)	G6PD deficit YES (*n* = 18)	Diabetes main effect	G6PD main effect	Interaction tern
Age (years)	50.1 ± 13.5	50.1 ± 8.6	62.3 ± 11.4	64.7 ± 11.1	–	–	–
Men (%)	41.8	39.3	52.6	33.3			
G6PD activity	1.2 ± 0.4	0.3 ± 0.3	1.2 ± 0.4	0.3 ± 0.3	–	.0001	–
BMI (Kg/m^2^)	23.9 ± 8.2	23.4 ± 7.3	27.4 ± 9.5	29.3 ± 8.4	.001	0.56	0.26
Waist circumference (cm)	87.2 ± 12.5	85.5 ± 11.9	99.5 ± 11.5	98.4 ± 12.4	.001	0.21	0.91
SBP (mmHg)	128.6 ± 19.0	127.7 ± 19.0	138.6 ± 18.4	144.2 ± 21.8	.05	0.51	0.22
DBP (mmHg)	79.7 ± 10.6	78.0 ± 10.1	82.5 ± 11.5	82.3 ± 10.4	0.71	0.33	0.74
HR (bpm)	66.7 ± 10.9	66.8 ± 10.7	69.5 ± 11.6	68.7 ± 13.9	0.07	0.81	0.75
Fasting glucose (mg/dl)	88.3 ± 11.1	87.0 ± 10.1	169.0 ± 50.1	178.2 ± 55.1	.001	.05	.01
LDL cholesterol (mg/dl)	135.3 ± 33.8	129.2 ± 33.1	131.2 ± 38.2	130.3 ± 33.6	0.13	0.37	0.61
HDL cholesterol (mg/dl)	65.4 ± 15.0	64.4 ± 14.8	61.1 ± 14.5	59.4 ± 12.6	.01	0.43	0.81
Triglycerides (mg/dl)	90.7 ± 54.2	84.7 ± 50.7	122.4 ± 65.7	120.8 ± 71.9	.001	0.55	0.8
Serum creatinine (mg/dl)	0.82 ± 0.22	0.82 ± 0.23	0.85 ± 0.26	0.80 ± 0.23	0.59	0.26	0.28
Hemoglobin (g/dl)	13.8 ± 1.5	13.5 ± 1.5	14.1 ± 1.4	13.5 ± 1.4	0.68	.05	0.47
AntiHT drugs (%)	11.9	14.1	39.5	38.9	.001	0.94	0.53
Anti diabetic drugs(%)	–	–	44.3	55.6	–	–	–
Lipid-lowering drugs (%)	2.9	2.3	10.5	11.1	.01	0.91	0.89
*PWV (m/sec)*							
Crude	7.2 ± 2.2	7.1 ± 2.0	9.2 ± 2.8	10.3 ± 2.7	001	0.16	.005
Age- and Glucose- adjusted*	7.3 ± 0.03	7.2 ± 0.1	7.2 ± 0.2	8.0 ± 0.4	0.19	0.22	.04
PWV/MBP	7.5 ± 2.1	7.4 ± 1.8	9..2 ± 2.7	10.1 ± 2.9	.001	0.16	.05
*Arterial aging (%)*							
HVA	5.0	5.7	1.8	0	.001	0.19	.05
Control	90.1	91.4	87.3	77.8			
EVA	4.9	2.9	10.9	22.2			
Adiponectin (mg/dl)	2.8 ± 2.0	2.9 ± 2.1	2.5 ± 1.8	2.8 ± 1.6	.05	0.55	0.78
Leptin (ng/ml)	8332 ± 8210	7269 ± 7012	10,553 ± 9974	11,532 ± 9745	.05	0.89	0.37
HsCRP (mg/ml)	2.6 ± 3.6	2.7 ± 3.7	4.1 ± 5.0	5.7 ± 6.1	.001	0.11	0.13
IL 6 (pg/ml)	3.1 ± 2.5	3.1 ± 2.5	4.2 ± 3.0	6.9 ± 5.0	.001	.001	.001

*Least square means ± Standard error

Although, PWV increases with both age and higher glucose levels, after adjustment for age and glucose, differences in PWV and IL6 in diabetics with and without G6PD deficiency remained significant.

## Conclusions

This is the first study to demonstrate that G6PD deficiency in diabetics, but not non-diabetics, is associated with higher plasma IL6 levels and stiff arteries.

The observation of increased IL6 levels deserves further comment. Adipose tissue inflammation has more and more emerged as a critical path with complex regulation [17, 18] leading to adipose tissue and, then, to systemic insulin resistance—a key step in the onset of type 2 diabetes mellitus [19]. The hypothesized time-dependent alterations start with adipocyte hypertrophy, followed by increased levels of macrophage stimulating factor and eventually with higher IL-6 levels in plasma [19, 20]. Therefore, IL6 circulating levels are a facet of mechanistically relevant alteration occurring at adipose tissue level.

Notably, genetic loci recently identified are associated with insulin resistance at a lower level of adiposity [21] and promote endothelium-specific insulin resistance [22].

Endothelial insulin-resistance may trigger alteration in vascular extracellular matrix composition and increased oxidative stress of its components [23]. Alteration in vascular extracellular matrix composition is emerging as key determinant of greater arterial stiffness and accelerated arterial aging [24].

Of note, G6PD deficiency is further reduced by nonenzymatic glycation in states of hyperglycemia, creating a self-reinforcing loop [25], reducing endothelial nitric oxide bioavailability [26, 27] and increasing oxidative stress with activation of proinflammatory pathways in G6PD [7], eventually resulting in arterial stiffening and remodelling [28].

Future studies are needed to clarify tissue-specific G6PD contribution to arterial stiffening in diabetic subjects, and its possible role for the development of new therapeutic agents able to reduce the cardiovascular burden of diabetes mellitus.
